# Adaptive phenotypic plasticity in malaria parasites is not constrained by previous responses to environmental change

**DOI:** 10.1093/emph/eoz028

**Published:** 2019-09-28

**Authors:** Philip L G Birget, Petra Schneider, Aidan J O’Donnell, Sarah E Reece

**Affiliations:** Institute of Evolutionary Biology and Institute of Immunology and Infection Research, School of Biological Sciences, University of Edinburgh, Charlotte Auerbach Road, Edinburgh EH9 3FL, UK

**Keywords:** life history theory, Plasmodium, resource allocation trade-off, phenotypic plasticity, survival

## Abstract

**Background and objectives:**

Phenotypic plasticity enables organisms to maximize fitness by matching trait values to different environments. Such adaptive phenotypic plasticity is exhibited by parasites, which experience frequent environmental changes during their life cycle, between individual hosts and also in within-host conditions experienced during infections. Life history theory predicts that the evolution of adaptive phenotypic plasticity is limited by costs and constraints, but tests of these concepts are scarce.

**Methodology:**

Here, we induce phenotypic plasticity in malaria parasites to test whether mounting a plastic response to an environmental perturbation constrains subsequent plastic responses to further environmental change. Specifically, we perturb red blood cell resource availability to induce *Plasmodium chabaudi* to alter the trait values of several phenotypes underpinning within-host replication and between-host transmission*.* We then transfer parasites to unperturbed hosts to examine whether constraints govern the parasites’ ability to alter these phenotypes in response to their new in-host environment.

**Results:**

Parasites alter trait values in response to the within-host environment they are exposed to. We do not detect negative consequences, for within-host replication or between-host transmission, of previously mounting a plastic response to a perturbed within-host environment.

**Conclusions and implications:**

We suggest that malaria parasites are highly plastic and adapted to adjusting their phenotypes in response to the frequent changes in the within-host conditions they experience during infections. Our findings support the growing body of evidence that medical interventions, such as anti-parasite drugs, induce plastic responses that are adaptive and can facilitate the survival and potentially, drug resistance of parasites.

**Lay Summary:**

Malaria parasites have evolved flexible strategies to cope with the changing conditions they experience during infections. We show that using such flexible strategies does not impact upon the parasites’ ability to grow (resulting in disease symptoms) or transmit (spreading the disease).

## INTRODUCTION

The ability to alter aspects of phenotype in response to changes in environmental conditions (phenotypic plasticity) is commonly observed in multicellular organisms [1–3]. But the notion that phenotypic plasticity is central to the fitness of parasites has been met with controversy in medicine and parasitology [4, 5]. This is likely due to the longstanding assumption that parasites, since they live inside their hosts, are sheltered from the rapidly changing conditions of the exterior environment, such as weather, temperature and predators, light and dark, and that there is a high autocorrelation in conditions experienced during infections and between hosts [6, 7]. An alternative view is that parasites are actually confronted with frequent and fast changing within-host conditions during each infection, as well as variation in conditions experienced in different hosts (particularly for parasites with complex, multi-host, life cycles). A number of within-host environmental factors vary during infections and across hosts, including the availability of resources, mating opportunities, immune responses, intensity of competition with co-infecting parasites, medical interventions and daily rhythms in host physiology. Parasites, stemming from diverse phyla, appear to cope with the challenges and opportunities arising from variable within-host conditions using phenotypic plasticity in a wide variety of traits. For example, the nematode *Strongyloides ratti* modifies its (generally stable) transcriptome in immunized hosts to avoid expulsion [8]. The trematode *Coitocaecum parvum* adopts a three-host or a two-host lifecycle depending on the availability of appropriate definitive hosts [9]. Bacteriophages vary their lysis/lysogeny decision, and malaria parasites their sex ratio and reproductive effort, in response to co-infecting conspecific genotypes [10, 11].

Like all traits, the evolution and expression of phenotypic plasticity is assumed to be subject to costs, constraints and trade-offs. These concepts are often hard to define and differentiate, and so are rarely examined empirically [12], especially for parasites/pathogens [5]. Costs of plasticity can be thought of as phenomena that reduce the fitness of a plastic genotype compared to a non-plastic genotype expressing the same trait value [12, 13]. Constraints of plasticity can be defined as phenomena that prevent organisms from reaching the optimal phenotype that a given environment demands [13]. For example, plasticity of many developmental traits is constrained by a window of sensitivity, i.e. a trait is only responsive to environmental change during early development and becomes fixed for the remaining lifetime of the individual. Thus, there is a risk that a trait is beneficial in early life but may become suboptimal at a later point because environmental conditions are likely to change. Such constraints include so-called ‘trans-host effects’ (in analogy with transgenerational effects on genetic expression), which occur when, for example, environmental conditions in the current host affect the behaviour of a parasite in the next host [14–17]. Analogous effects could occur during dynamic infections if parasites are unable to keep up with rapid changes in immune responses and resource availability. Thus, if parasites rely on phenotypic plasticity to optimize host exploitation and maximize transmission, then the history of hosts/cells/within-cell conditions that parasites have experienced could constrain the trait values of adaptive plastic responses to future conditions as well as reduce within-host replication, shorten infection duration or lower the rate of between-host transmission [18–20].

Here, we use the rodent malaria parasite *Plasmodium chabaudi* to explore the consequences of deploying plasticity. We do not test for inherent costs of plasticity because that would require comparing plastic and non-plastic parasites. Because non-plastic *P.chabaudi* genotypes do not exist, we use a different approach and test whether the effect of plasticity in response to an environmental change effects future responses and parasite performance.

Malaria parasites replicate asexually in the vertebrate host’s red blood cells (RBC), but need to produce non-replicating sexual stages (‘gametocytes’) for between-host transmission, resulting in a trade-off between the allocation of resources to within-host replication and between-host transmission [21]. Malaria parasites adaptively adjust the proportion of each cohort of asexually replicating stages that commits to gametocyte development (‘conversion rate’) in response to within-host environmental conditions, including resource availability, competition with co-infecting parasites and antimalarial drug treatment [22–31]. Plasticity in other traits that underpin within-host replication also exists. For example, *Plasmodium berghei* reduces the number of progeny (merozoites) produced per asexual stage (‘burst size’) in response to caloric restriction of the host [32], and *P.chabaudi* increases burst size in anaemic hosts [33]. We harness the plastic responses of *P.chabaudi* to host anaemia [33] to investigate the impact of previous plastic responses on trait values for conversion rate, burst size and asexual replication. Specifically, we exposed parasites to either control hosts or anaemic hosts, to generate parasites with a different history of plasticity-driven trait values. Subsequently, we mimic a rapid change of the within-host environment by transferring parasites from each control host and each anaemic host to non-anaemic hosts that serve as a common garden, i.e. parasites from both initial environments experience the same final within-host environment. An impact of the history of past within-host conditions and/or previously mounting a plastic response (beyond the reduction of asexual parasite densities), should manifest as a difference in trait values in the common garden hosts.

## METHODS

**Figure 1.**
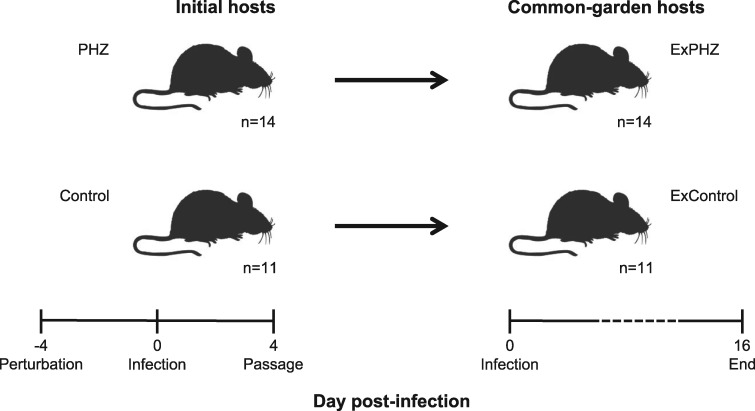
Experimental setup. We treated initial hosts with 30 mg/kg of PHZ to generate anaemia, or with PBS (control) on day–4 PI, and infected them with 5 × 10^6^*P.chabaudi* AS infected RBCs on day 0 PI. On day 4 PI we transferred 5 × 10^6^ infected RBCs from each initial host into common garden hosts by one-to-one passage, and we followed these infections for 16 days. In all sets of hosts, we measured the daily densities (dynamics) of asexual stages and gametocytes, burst size (on day 4 PI) and we estimated conversion rates for the CG hosts

The experiment comprised of two sets of hosts (Fig. 1). Parasites in initial hosts experienced a within-host environment of untreated hosts or of phenylhydrazine (PHZ) treated, anaemic hosts. Parasites were then transferred from the initial hosts to untreated, common garden hosts. We monitored the dynamics of asexual parasites and gametocytes, and burst size (number of merozoites/schizont) in the initial hosts to confirm parasites adopt different trait values due to mounting plastic responses to anaemia, and then monitored traits in the common garden hosts.

### Parasites and hosts

We obtained C57BL/6 female mice (aged 6–8 weeks) in-house (University of Edinburgh) and the *P.chabaudi* clone AS (AS12537) from the Edinburgh Malaria reagent repository. All animal care was in accordance with institutional and UK Home Office guidelines. *P.chabaudi* was isolated from African thicket rats (*Grammomys poensis*, previously called *Thamomnys rutilans*) in Central Africa [34], and *Mus musculus* is a natural host for some rodent malaria species [35]. After cloning, genotype AS has been cryopreserved and undergone regular transmission through mosquitoes to maintain its wild type phenotype [36]. We treated initial hosts 4 days before infection (day–4 post-infection (PI)) with a single intraperitoneal injection of either 30 mg/kg of PHZ dissolved in phosphate-buffered saline (PHZ, *n* = 14) or phosphate-buffered saline (Control, *n* = 11). PHZ causes anaemia by provoking the premature clearance of mature RBCs, leading to the release of immature RBCs (reticulocytes) into the blood as shown in [23] through an erythropoietin-mediated feedback loop [37].

All initial hosts received an intravenous injection of 5 × 10^6^ AS-parasitized RBCs on day 0 PI. On day 4 PI, we transferred 5 × 10^6^ parasitized RBCs from each initial host into a randomly chosen, untreated, common garden host (CG hosts, *n* = 25) by a one-to-one intravenous passage. Given that all hosts were inbred and age and sex matched, our design simulates a rapid change in within-host conditions (without the confounding effects of age of infection). We chose untreated hosts for our common garden hosts because *P.chabaudi* replicates faster in PHZ-treated compared to untreated hosts [23, 33]. This suggests that untreated hosts may provide a harsher environment for the parasites, in which constraints may be more easily observed. We designate the common garden hosts that received their parasites from a PHZ-treated initial host as an ‘ExPHZ’ infection (*n* = 14) and those that received their parasites from a control initial host as an ‘ExControl’ infection (*n* = 11). We monitored infections in the initial hosts from days 1 to 4 PI and from days 1 to 16 PI in the common garden hosts. Our design thus relies on three assumptions: first, that parasites encounter different within-host environments between PHZ-treated and control initial hosts, and second, that parasites exhibit different phenotypes between PHZ-treated and control initial hosts. Third, we assume that different phenotypes between the PHZ-treated and control initial hosts do not result from genetic adaptation to our reticulocyte-rich anaemic hosts. Such adaptation is extremely unlikely in our experiment for several non-mutually exclusive reasons: (i) parasites were expanded from cryopreservation in untreated hosts that are not reticulocyte-rich anaemic hosts. Any favourable variants would therefore have to be selected for in only three replication cycles in our initial hosts; (ii) for a single mutation to underpin such evolution, the highest possible estimate for a mutation to arise and expand in this time would result in the ‘adapted parasites’ to be at approx. 0.01% of the total parasite population; (iii) serial passage can result in genetic evolution in *P.chabaudi* but this generally requires 11–20 passages of infections each lasting at least 7 days [38–40]; (iv) plasticity may constrain genetic evolution because the ability to adjust trait values to suit the new environment buffers against fitness loss due to environmental changes, which in turn reduces the strength of selection on fixed traits [41]; (v) any adaptation to specifically infect reticulocytes is unlikely to have provided significant fitness benefits because even in PHZ-treated hosts, reticulocytes comprise a minority of the total RBC population.

### Data collection and analysis

For all initial and common garden hosts, we measured RBC density and RBC age structure (the proportion of RBCs that are immature RBCs; i.e. reticulocytes), the proportion of RBCs infected with asexual stages and gametocyte density daily, with the exception of day 1 PI gametocyte density in the initial hosts. We quantified RBC density from 2 µl blood by flow cytometry (Beckman Coulter), and made thin blood smears to count the ratio of mature (normocytes) to immature (reticulocytes) RBCs, as well as the proportion of asexual stage infected RBCs, by microscopy. We quantified gametocyte densities from 10 μl blood samples by reverse-transcriptase quantitative PCR [42] as described previously [23]. We assessed burst size on day 4 PI as the number of merozoites counted from the first 30 mature schizonts observed on blood smears taken shortly before schizogony (from a random subset of *n* = 4 per treatment for the initial hosts, and for all common garden host infections).

We characterized the within-host environment based on the density of total RBC (i.e. the combined density of normocytes and reticulocytes) and their age structure (i.e. the proportion of RBCs that are reticulocytes). We calculated daily asexual parasite densities by multiplying RBC density with the proportion of infected RBCs, and used daily asexual parasite densities to derive replication rate ($\mathrm{replication}\mathrm{rate}\mathrm{day}t=\frac{\mathrm{asexual}\mathrm{parasite}\mathrm{density}\mathrm{day}(t+1)}{\mathrm{asexual}\mathrm{parasite}\mathrm{density}\mathrm{day}(t)}$. It was only possible to calculate conversion rates for infections of CG hosts since data from a longer infection duration than in the initial hosts is required to use a state-of-the-art spline-based model, developed by Greischar *et al.* [43].

All calculations and statistical analysis were carried out using R version 3.2.4. To analyse the dynamics of host and parasite traits during the infections, we used linear mixed effect models including treatment, day PI and their interaction as fixed effects, and mouse identification number as a random effect to account for repeated measures. For the analyses of burst size data we used linear mixed effect models including treatment as a fixed effect, and mouse identification number as a random effect to account for repeated measures. We minimized nested models using maximum likelihood deletion tests, and we transformed response variables to meet assumptions of homogeneity of variance (proportion of reticulocytes and replication rates were square root transformed, whilst asexual parasite density, gametocyte density and conversion rate were log_10_ transformed adding half a counting unit to all data to deal with zero values according to [44]). To compare infections in initial with common garden hosts, we used only data covering the same sample time points in both data sets. We first used the above approach to verify that there was a difference between the four host types, and subsequently compared the four host type model with a model that groups untreated initial hosts with the common garden hosts (i.e. PHZ vs Control+ExControl+ExPHZ) to confirm that the PHZ is the outlying group. We present significant test statistics in the text, whilst full statistical details are shown in Supplementary Table S1.

## RESULTS

Our experiment required comparing plasticity in parasite traits for parasites originating from either untreated (Control) or anaemic, PHZ-treated initial hosts, after being placed in the same environment: untreated, non-anaemic common garden hosts (ExControl, ExPHZ). First, we confirm the assumption that parasites experience different environments in Control and PHZ-treated initial hosts. Second, we confirm that parasites adopt different phenotypes in Control and PHZ-treated initial hosts. Third, we compare the trait values of parasites—stemming from different types of initial hosts—in common garden hosts (ExControl vs ExPHZ), to test for effects of previous plasticity, i.e. the trait values adopted in initial hosts.

### RBC environments and parasite traits vary between PHZ-treated and control initial hosts

**Figure 2.**
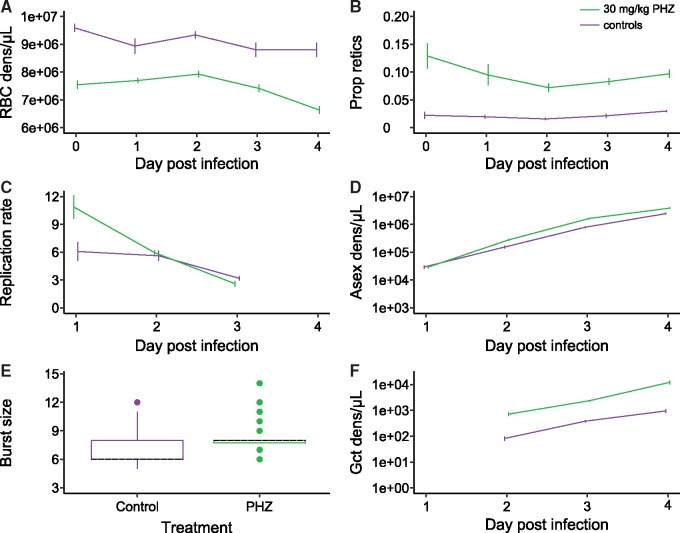
The within-host environment and parasite traits differ between PHZ-treated (PHZ, green) and untreated (Control, purple) initial hosts. RBC densities are lower (RBC dens, **A**) and the proportion of RBCs that are reticulocytes are higher (Prop retics, **B**) in PHZ-treated initial hosts. Asexual replication rates were higher in PHZ-treated initial hosts on day 1 PI (**C**), resulting in higher asexual densities on days 2–4 PI (Asex dens, **D**). Burst sizes, measured as the number of merozoites per schizont on day 4 PI (**E**), and gametocyte densities (Gct dens, **F**) were also higher in PHZ-treated initial hosts. Graphs show mean ± SEM (A–D, F) or median (black dashed) and 25–75 percentiles, with whiskers 1.5 times the interquartile range and dots representing outliers (E)

PHZ treatment generated anaemia, which was maintained in the PHZ-treated initial hosts throughout days 0–4 PI, as measured by a decrease in RBC densities (treatment * dayPI interaction: χ^2^ (4) = 13.92, *P* = 0.0076) and an increased proportion of RBCs that were reticulocytes (treatment effect: χ^2^(1)=44.31, *P* < 0.0001) (Fig. 2A and B).

Parasites adopted different trait values in control and PHZ-treated initial hosts in the manners described previously [23, 33]. First, daily asexual replication rates varied between treatments (treatment * dayPI interaction: χ^2^ (2) = 15.96, *P* = 0.0003), with replication rates in PHZ-treated hosts higher than in untreated hosts on day 1 PI (Fig. 2C). This results in differences between treatments for the dynamics of asexual densities during days 1–4 PI (treatment * dayPI interaction: χ^2^ (3) = 21.51, *P* < 0.0001), with higher asexual parasite densities in PHZ-treated initial hosts than in control initial hosts during days 2–4 PI (Fig. 2D). Second, parasites in PHZ-treated initial hosts produced, on average, 1.15 times more merozoites per schizont than parasites in control initial hosts (χ^2^ (1) = 12.73, *P* = 0.0004, Fig. 2E). Third, the dynamics of gametocytes densities differed between treatments (treatment * dayPI interaction: χ^2^ (2) = 9.92, *P* = 0.0070), with gametocyte densities in PHZ-treated initial hosts higher than those in control initial hosts (Fig. 2F).

### Trait values in the common garden hosts

**Figure 3.**
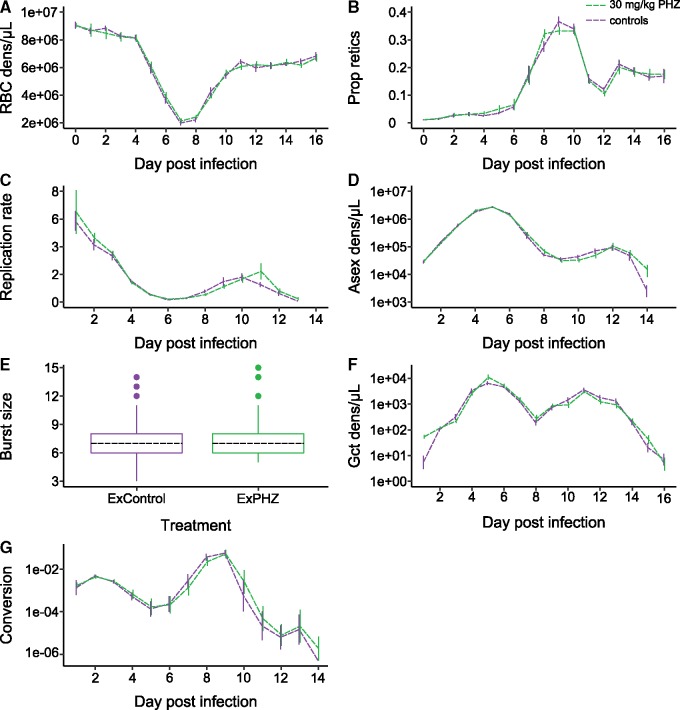
The within-host environment and parasite traits do not differ between common garden hosts (ExPHZ, green and ExControl, purple). We observed no significant differences in RBC densities (RBC dens, **A**), the proportion of RBCs that are reticulocytes (Prop retics, **B**), asexual replication rates (**C**), asexual parasite densities (Asex dens, **D**) or burst sizes, measured as the number of merozoites per schizont, on day 4 PI (**E**) between ExPHZ and ExControl hosts. Gametocyte densities are higher in ExPHZ compared to ExControl hosts on day 1 PI only because these gametocytes originate from infections in the initial hosts (Gct dens, **F**) and conversion rates were not significantly different between common garden hosts (G). Graphs show mean ± SEM (A–D, F–G) or median (black dashed) and 25–75 percentiles, with whiskers 1.5 times the interquartile range and dots representing outliers (E)

The dynamics of RBC densities (treatment: χ^2^ (1) = 0.002, *P* = 0.9617) and of proportion of reticulocytes (treatment: χ^2^ (1) = 0.001, *P* = 0.9719) were not significantly different between the two groups of untreated common garden hosts (ExPHZ and ExControl) for days 0–16 PI (Fig. 3A and B). For the time frame during which data were collected in the initial hosts (days 0–4 PI), the RBC environment did not significantly differ between the untreated common garden hosts (ExPHZ and ExControl) and the control initial hosts (RBC density χ^2^ (10) = 9.74, *P* = 0.4634; proportion reticulocytes χ^2^ (10) = 12.07, *P* = 0.2804). Thus, the assumption that parasites in control initial hosts and all common garden hosts experienced the same RBC environment was met.

We did not detect any significant differences in asexual stage traits between ExPHZ and ExControl parasites (Fig. 3C–E). This includes the dynamics of replication rate (Fig. 3C, treatment: χ^2^ (1) = 0.27, *P* = 0.6059) and of asexual densities (Fig. 3D, treatment: χ^2^ (1) = 0.03, *P* = 0.8625), and day 4 PI burst sizes (Fig. 3E, treatment: χ^2^ (1) = 0.058, *P* = 0.8103). We did observe a difference in gametocyte dynamics between ExPHZ and ExControl parasites (Fig. 3F, treatment * dayPI interaction: χ^2^ (15) = 45.24, *P* < 0.0001), driven by gametocyte densities at the start of infections. Gametocytes on day 1 PI are likely carried over from the initial hosts as well as originating from the conversion decision of the asexual parasites in the inoculum used to infect the common garden hosts, and therefore reflect the RBC environments of the initial hosts rather than reflecting the performance of parasites in common garden hosts. When excluding day 1 PI from the analysis, neither the treatment * dayPI interaction (χ^2^ (14) = 13.79, *P* = 0.4654) or treatment (χ^2^ (1) = 0.0008, *P* = 0.977) were significant (Fig. 3F). Furthermore, daily conversion rates, i.e. the proportional investment of asexual parasites into gametocytes, were not significantly different between ExPHZ and ExControl parasites (Fig. 3G, treatment: χ^2^ (1) = 0.18, *P* = 0.6748).

## DISCUSSION

We asked whether mounting phenotypically plastic responses to a perturbation of the within host environment imposes longer-term constraints on future plastic responses. Harnessing the diverse phenotypically plastic traits expressed by *P.chabaudi* malaria parasites in response to host anaemia revealed no such effects. Specifically, parasites transferred from anaemic initial hosts exhibited trait values that were not significantly different to those of parasites transferred from control initial hosts. We assume that plasticity in the traits investigated here are at least in part under parasite control. In principle, variation in burst size, replication rate or conversion could be host-imposed. For example, if reticulocytes are more nutritious (e.g. if they contain more LysoPC [45]), the age structure of RBCs could directly influence replication rate. However, evidence suggests that plasticity in parasite traits is at least in part controlled by parasites. For example, the anaemia-driven increase in burst size and conversion rate is not directly related to growth inside reticulocytes but seems to be a response to the presence of reticulocytes per se [23, 28]. Furthermore, that conversion rate is altered adaptively in response to a number of within-host variables, exhibits genetic variation, and cannot be explained by differential mortality, strongly suggests parasites control conversion rate [25, 28, 46].

The age composition of RBCs is expected to determine the repertoire of invasion receptors expressed by merozoites [47, 48] because reticulocytes and normocytes offer different ligands. This could constrain the replication rate of parasites coming from a reticulocyte rich, anaemic host if they experience a delay in switching to the repertoire required in the reticulocyte rare environment of a common garden control host. Our data provide no evidence of such a constraint because the dynamics of asexual stages did not vary between the two groups of common garden hosts. Whereas a reduction in replication rate could be offset by lowering conversion rate and/or increasing burst size, we did not detect any differences in these traits either. Furthermore, gametocyte densities are higher in PHZ-treated initial hosts on day 2 PI, whilst originating from the same asexual densities as parasites in the control hosts. Although we did not directly quantify conversion rates in the initial hosts, this implies conversion rates were increased in PHZ-treated hosts, as shown previously [23]. Any costs/constraints related to such elevated conversion rates did not affect the replicative capacity of asexual stages or future conversion to gametocytes in common garden hosts. Whilst conversion rate is the plastically adjusted trait, the resulting density of gametocytes is better proxy for short-term transmission probability (i.e. parasite fitness). We found no evidence of fitness costs in terms of lower gametocyte densities in parasites stemming from anaemic initial hosts.

The absence of a detectable effect of previous plasticity may have several, non-mutually exclusive, explanations. (i) It is possible that our experimental design was unable to detect small effects due to low power. (ii) Parasites may readily change reticulocyte/normocyte invasion ligands upon arrival in the common garden hosts. (iii) The repertoire of invasion ligands expressed by *P.chabaudi* may always be diverse enough for efficient invasion independent of RBC age structure [33]. (iv) If asexual density is viewed as a fitness proxy, the lower density in control initial hosts on the day of transfer could be viewed as a cost, but this was ameliorated by initiating infections in common garden hosts with a fixed number of parasites. This approach controls the impact of inoculum dose on infection dynamics, allowing us to test for effects of ‘quality’ rather than ‘quantity’ of parasites. (v) Costs/constraints may only manifest as trans-host, or host-vector effects, which we have not tested for. Although we use a sequence of hosts (initial and common garden hosts), we transferred parasites by blood passage, rather than by mosquito (which is the natural route for transmission), to simulate the changes in RBC density and age structure experienced during infections. (vi) The common garden environment provided by naïve hosts was not stressful enough to elicit constraints. For example, Steinger *et al.* report that sib-families of the plant *Sinapis arvensis* that show greater plasticity in specific leaf area also suffer larger fitness costs in a shady environment than in full light conditions [49], and plasticity in internode length in *Ranunculus reptans* is more costly in competitive than under benign conditions [50]. Intuitively, naïve hosts appear to be a benign environment. However, given that parasites replicate faster in PHZ-treated compared to untreated hosts [23, 33], this suggests that untreated hosts are the harsher environment, but perhaps not harsh enough. (vii) We tested a single parasite genotype to balance ethical considerations with testing for proof of principle. It is possible that our results may not be easily generalized to other genotypes. However, we generally see qualitatively similar responses to environmental perturbations across the available *P.chabaudi* genotypes. Genotype by environment interactions are sometimes observed but these result in quantitative, rather than qualitative, differences in the genotypes’ responses to environmental change (e.g. [11, 23, 25, 33]). Further, across the *P.chabaudi* clone bank, the genotype we chose (AS) displays an intermediate level of plasticity in the traits we studied (a.o. [23]), so we expect it is representative of the population. (viii) We only exposed parasites to a single environmental change (anaemic to control hosts) and constraints and limits may only emerge after parasites experience a longer sequence of different within-host conditions. If costs and constraints are mainly exposed in stressful environments [51], future work could examine parasites in common garden hosts that involve competition with con-specific genotypes, in semi-immune hosts or during drug treatment.

One of the preconditions for plasticity to evolve is that its costs, relative to the negative consequences of suboptimal trait values, must be low [12, 13]. For parasites that live in variable environments or that require extensive changes to their traits, selection may have honed rapid responses and minimized costs/constraints. For example, the parasitic nematode *Heligmosomoides polygyrus* increases expression of two immunomodulatory genes in hosts mounting an inflammatory response, but readily reduces expression when in hosts without inflammatory responses, even after experiencing upregulated inflammation for four parasite generations [52]. If costs and constraints do exist, they may only be exposed in stressful environments [51]. Thus future work could examine parasites in common garden hosts that involve competition with con-specific genotypes, in semi-immune hosts, or during drug treatment. By using a single parasite genotype (to avoid any confounding effects of genetic variation), we assume that because conversion rates vary day-to-day (Fig. 3G), parasites in both types of initial host and in common garden hosts are paying the costs of environmental sensing. The standing costs of plasticity are thought to be low, but are hard to measure [12, 13]; future work that selects for parasites with a fixed conversion rate might allow these costs to be measured. Finally, the generality of our results can be confirmed by extending experiments to include multiple genotypes and species of malaria parasites.

Here we show that parasites are able to rapidly adjust fitness-related life history traits to match changes in conditions experienced in the within-host environment. Mechanisms controlling environmental sensing in malaria parasites are only partly understood (e.g. [32]), but evidence for epigenetic mechanisms governing commitment to gametocytes is accumulating rapidly [53–56]. Thus, in the context of conversion at least, our findings suggest these epigenetic mechanisms quickly respond to environmental change. The capacity for rapid and generally appropriate responses to environmental change may play an underappreciated role in treatment failure and the evolution of resistance [41]. For example, plasticity in conversion allows parasites to find the best balance between short- and long-term transmission after drug treatment [28]. Given that different types of environmental change demand different trait values, could parasites be forced to make suboptimal decisions by exposing them to several such environmental changes, simultaneously?

## Supplementary Material

eoz028_Supplementary_Data

## References

[1] Scheiner SM. Genetics and evolution of phenotypic plasticity. Annu Rev Ecol Sys 1993;24:35–68.

[2] Schlichting CD, Smith H. Phenotypic plasticity: linking molecular mechanisms with evolutionary outcomes. Evol Ecol 2002;16:189.

[3] Pigliucci M. Phenotypic Plasticity: Beyond Nature and Nurture. Baltimore: Johns Hopkins University Press, 2001.

[4] Kochin BF, Bull JJ, Antia R. Parasite evolution and life history theory. PLoS Biol 2010;8:e1000524. 20976100 10.1371/journal.pbio.1000524 PMC2957397

[5] Thomas F, Brown SP, Sukhdeo M et al Understanding parasite strategies: a state-dependent approach? Trends Parasitol 2002;18:387–90. 12377254 10.1016/s1471-4922(02)02339-5

[6] Sukhdeo MV, Sukhdeo SC. Optimal habitat selection by helminths within the host environment. Parasitology 1994;109:S41–55. 7854851 10.1017/s0031182000085073

[7] Viney M, Cable J. Macroparasite life histories. Curr Biol 2011;21:R767–R774. 21959167 10.1016/j.cub.2011.07.023

[8] O'Meara H, Barber R, Mello LV. Response of the *Strongyloides ratti* transcriptome to host immunological environment. Int J Parasitol 2010;40:1609–17. 20673765 10.1016/j.ijpara.2010.06.005

[9] Lagrue C, Poulin R. Life cycle abbreviation in the trematode *Coitocaecum parvum*: can parasites adjust to variable conditions? J Evol Biol 2007;20:1189–95. 17465928 10.1111/j.1420-9101.2006.01277.x

[10] Leggett HC, Benmayor R, Hodgson DJ et al Experimental evolution of adaptive phenotypic plasticity in a parasite. Curr Biol 2013;23:139–42. 23246405 10.1016/j.cub.2012.11.045

[11] Reece SE, Drew DR, Gardner A. Sex ratio adjustment and kin discrimination in malaria parasites. Nature 2008;453:609–14. 18509435 10.1038/nature06954 PMC3807728

[12] Auld JR, Agrawal AA, Relyea RA. Re-evaluating the costs and limits of adaptive phenotypic plasticity. Proc Biol Sci 2010;277:503–11. 19846457 10.1098/rspb.2009.1355 PMC2842679

[13] Murren CJ, Auld JR, Callahan H et al Constraints on the evolution of phenotypic plasticity: limits and costs of phenotype and plasticity. Heredity 2015;115:293–301. 25690179 10.1038/hdy.2015.8 PMC4815460

[14] Little T, Birch J, Vale P et al Parasite transgenerational effects on infection. Evol Ecol Res 2007;9:459–69.

[15] Tseng M. Interactions between the parasite's previous and current environment mediate the outcome of parasite infection. Am Nat 2006;168:565–71. 17004228 10.1086/507997

[16] Benesh DP, Chubb JC, Parker GA. Complex life cycles: why refrain from growth before reproduction in the adult niche? Am Nat 2013;181:39–51. 23234844 10.1086/668592

[17] Costa G, Gildenhard M, Eldering M et al Non-competitive resource exploitation within mosquito shapes within-host malaria infectivity and virulence. Nat Commun 2018;9:3474. 30150763 10.1038/s41467-018-05893-z PMC6110728

[18] Hakalahti T, Bandilla M, Valtonen ET. Delayed transmission of a parasite is compensated by accelerated growth. Parasitology 2005;131:647–56. 16255823 10.1017/S0031182005008279

[19] Gleichsner AM, Cleveland JA, Minchella DJ. One stimulus-two responses: host and parasite life-history variation in response to environmental stress. Evolution 2016;70:2640–6. 27596485 10.1111/evo.13061

[20] Babayan SA, Read AF, Lawrence RA et al Filarial parasites develop faster and reproduce earlier in response to host immune effectors that determine filarial life expectancy. PLoS Biol 2010;8:e1000525. 20976099 10.1371/journal.pbio.1000525 PMC2957396

[21] Carter LM, Kafsack BF, Llinas M et al Stress and sex in malaria parasites: why does commitment vary? Evol Med Public Health 2013;2013:135–47. 24481194 10.1093/emph/eot011 PMC3854026

[22] Trager W, Gill GS, Lawrence C et al *Plasmodium falciparum*: enhanced gametocyte formation in vitro in reticulocyte-rich blood. Exp Parasitol 1999;91:115–8. 9990338 10.1006/expr.1998.4347

[23] Birget PLG, Repton C, O'Donnell AJ et al Phenotypic plasticity in reproductive effort: malaria parasites respond to resource availability. Proc Biol Sci 2017;284:1860. 10.1098/rspb.2017.1229 PMC5563815 28768894

[24] Cameron A, Reece SE, Drew DR et al Plasticity in transmission strategies of the malaria parasite, *Plasmodium chabaudi*: environmental and genetic effects. Evol Appl 2013;6:365–76. 23467678 10.1111/eva.12005 PMC3586624

[25] Pollitt LC, Mideo N, Drew DR et al Competition and the evolution of reproductive restraint in malaria parasites. Am Nat 2011; 177:358–67. 21460544 10.1086/658175 PMC3939351

[26] Bousema T, Drakeley C. Epidemiology and infectivity of *Plasmodium falciparum* and *Plasmodium vivax* gametocytes in relation to malaria control and elimination. Clin Microbiol Rev 2011;24:377–410. 21482730 10.1128/CMR.00051-10 PMC3122489

[27] Carter LM, Schneider P, Reece SE. Information use and plasticity in the reproductive decisions of malaria parasites. Malar J 2014;13:115. 24670151 10.1186/1475-2875-13-115 PMC3986881

[28] Schneider P, Greischar MA, Birget PLG et al Adaptive plasticity in the gametocyte conversion rate of malaria parasites. PLoS Pathog 2018;14:e1007371. 30427935 10.1371/journal.ppat.1007371 PMC6261640

[29] Birget PLG, Greischar MA, Reece SE et al Altered life history strategies protect malaria parasites against drugs. Evol Appl 2018;11:442–55. 29636798 10.1111/eva.12516 PMC5891063

[30] Peatey CL, Skinner-Adams TS, Dixon MW et al Effect of antimalarial drugs on *Plasmodium falciparum* gametocytes. J Infect Dis 2009;200:1518–21. 19848586 10.1086/644645

[31] Reece SE, Ali E, Schneider P et al Stress, drugs and the evolution of reproductive restraint in malaria parasites. Proc Biol Sci 2010;277:3123–9. 20484242 10.1098/rspb.2010.0564 PMC2982055

[32] Mancio-Silva L, Slavic K, Grilo Ruivo MT et al Nutrient sensing modulates malaria parasite virulence. Nature 2017; 547:213–6. 28678779 10.1038/nature23009 PMC5511512

[33] Birget PLG, Prior KF, Savill NJ et al Plasticity and genetic variation in traits underpinning asexual replication of the rodent malaria parasite *Plasmodium chabaudi*. Malaria J 2019;18:222. 10.1186/s12936-019-2857-0 PMC6604315 31262304

[34] Killick-Kendrick R. Taxonomy, Zoogeography and Evolution, in Rodent Malaria. New York: Academic Press, 1978.

[35] Boundenga L, Ngoubangoye B, Ntie S et al Rodent malaria in Gabon: diversity and host range. Int J Parasitol Parasites Wildl 2019;10:117–24. 31453086 10.1016/j.ijppaw.2019.07.010 PMC6702409

[36] Spence PJ, Jarra W, Levy P et al Vector transmission regulates immune control of Plasmodium virulence. Nature 2013;498:228–31. 23719378 10.1038/nature12231 PMC3784817

[37] Savill NJ, Chadwick W, Reece SE. Quantitative analysis of mechanisms that govern red blood cell age structure and dynamics during anaemia. PLoS Comput Biol 2009;5:e1000416. 19557192 10.1371/journal.pcbi.1000416 PMC2694369

[38] Barclay VC, Sim D, Chan BHK et al The evolutionary consequences of blood-stage vaccination on the rodent malaria *Plasmodium chabaudi*. PLoS Biol 2012;10:e1001368. 22870063 10.1371/journal.pbio.1001368 PMC3409122

[39] Mackinnon MJ, Read AF. Immunity promotes virulence evolution in a malaria model. PLoS Biol 2004; 2:E230. 15221031 10.1371/journal.pbio.0020230 PMC434153

[40] Mackinnon MJ, Read AF. Selection for high and low virulence in the malaria parasite *Plasmodium chabaudi*. Proc Biol Sci 1999;266:741–8. 10331293 10.1098/rspb.1999.0699 PMC1689830

[41] Mideo N, Reece SE. Plasticity in parasite phenotypes: evolutionary and ecological implications for disease. Fut Microbiol 2012;7:17–20. 10.2217/fmb.11.134 22191443

[42] Wargo AR, de Roode JC, Huijben S et al Transmission stage investment of malaria parasites in response to in-host competition. Proc Biol Sci 2007;274:2629–38. 17711832 10.1098/rspb.2007.0873 PMC1975767

[43] Greischar MA, Mideo N, Read AF et al Quantifying transmission Investment in malaria parasites. PLoS Comput Biol 2016;12:e1004718. 26890485 10.1371/journal.pcbi.1004718 PMC4759450

[44] Yamamura K. Transformation using (x + 0.5) to stabilize the variance of populations. Res Popul Ecol 1999;41:229–34.

[45] Brancucci NMB, Gerdt JP, Wang C et al Lysophosphatidylcholine regulates sexual stage differentiation in the human malaria parasite *Plasmodium falciparum*. Cell 2017;171:1532–44.e15. 29129376 10.1016/j.cell.2017.10.020 PMC5733390

[46] Reece SE, Schneider P. Premature rejection of plasticity in conversion. Trends Parasitol 2018;34:633–4. 29945759 10.1016/j.pt.2018.06.004

[47] Khan SM, Jarra W, Preiser PR. The 235 kDa rhoptry protein of *Plasmodium (yoelii) yoelii*: function at the junction. Mol Biochem Parasitol 2001;117:1–10. 11551627 10.1016/s0166-6851(01)00333-4

[48] Preiser PR, Jarra W, Capiod T et al A rhoptry-protein-associated mechanism of clonal phenotypic variation in rodent malaria. Nature 1999;398:618–22. 10217144 10.1038/19309

[49] Steinger T, Roy BA, Stanton ML. Evolution in stressful environments II: adaptive value and costs of plasticity in response to low light in *Sinapis arvensis*. J Evol Biol 2003;16:313–23. 14635870 10.1046/j.1420-9101.2003.00518.x

[50] van Kleunen M, Fischer M. Constraints on the evolution of adaptive phenotypic plasticity in plants. New Phytol 2005;166:49–60. 15760350 10.1111/j.1469-8137.2004.01296.x

[51] Chevin LM, Hoffmann AA. Evolution of phenotypic plasticity in extreme environments. Philos Trans R Soc Lond B Biol Sci 2017;372:20160138. 28483868 10.1098/rstb.2016.0138 PMC5434089

[52] Guivier E, Lippens C, Faivre B et al Plastic and micro-evolutionary responses of a nematode to the host immune environment. Exp Parasitol 2017;181:14–22. 28733132 10.1016/j.exppara.2017.07.002

[53] Brancucci NMB, Bertschi NL, Zhu L et al Heterochromatin protein 1 secures survival and transmission of malaria parasites. Cell Host Microbe 2014;16:165–76. 25121746 10.1016/j.chom.2014.07.004

[54] Coleman BI, Skillman KM, Jiang RHY et al A *Plasmodium falciparum* histone deacetylase regulates antigenic variation and gametocyte conversion. Cell Host Microbe 2014;16:177–86. 25121747 10.1016/j.chom.2014.06.014 PMC4188636

[55] Poran A, Notzel C, Aly O et al Single-cell RNA sequencing reveals a signature of sexual commitment in malaria parasites. Nature 2017;551:95–9. 29094698 10.1038/nature24280 PMC6055935

[56] Sinha A, Hughes KR, Modrzynska KK et al A cascade of DNA-binding proteins for sexual commitment and development in Plasmodium. Nature 2014;507:253–7 24572359 10.1038/nature12970 PMC4105895

