# Supplementary material for: Adaptive phenotypic plasticity in malaria parasites is not constrained by previous responses to environmental change
Source: Evol Med Public Health. 2019 Sep 28;2019(1):190–8. doi: 10.1093/emph/eoz028 (PMC6805783; doi:10.1093/emph/eoz028)
Supplement: eoz028_Supplementary_Data [file eoz028_supplementary_data.docx]

**Supplementary data**

**Table S1.** Overview of statistical outcomes from linear mixed effect models including treatment (tr), day post infections (dayPI) and their interaction, as well as mouse identity as a random effect to account for repeated measures.

**RBC density dynamics**

Initial hosts, day 0-4 PI Common garden hosts, day 0-16 PI

tr*dayPI: χ^2^(4)=13.92 p=0.0076^a^ tr*dayPI: χ^2^(16)= 8.24 p= 0.9413

tr: χ^2^(1)= 0.002 p= 0.9617

dayPI: χ^2^(4)= 898.23 p< 2.2e-16 ^a^

**Proportion reticulocyte dynamics**

Initial hosts, day 0-4 PI Common garden hosts, day 0-16 PI

tr*dayPI: χ^2^(4)=5.02 p=0.2852 tr*dayPI: χ^2^(16)= 10.37 p= 0.8466

tr: χ^2^(1)= 44.31 p = 2.801e-11^a^ tr: χ^2^(1)= 0.001 p= 0.9719

dayPI: χ^2^(4)= 12.01 p = 0.0173 ^a^ dayPI: χ^2^(4)= 885.29 p< 2.2e-16 ^a^

**Replication rate dynamics**

Initial hosts, day 1-3 PI Common garden hosts, day 1-13 PI

tr*dayPI: χ^2^(2)=15.96 p=0.0003^a^ tr*dayPI: χ^2^(12)= 7.71 p= 0.8077

tr: χ^2^(1)= 0.27 p= 0.6059

dayPI: χ^2^(12)= 466.85 p< 2.2e-16^a^

**Asexual parasite dynamics**

Initial hosts, day 1-4 PI Common garden hosts, day 1-14 PI

tr*dayPI: χ^2^(3)=21.51 p=8.26e-05 ^a^ tr*dayPI: χ^2^(13)= 1.65 p= 0.9999

tr: χ^2^(1)= 0.03 p= 0.8625

dayPI: χ^2^(13)= 488.50 p< 2.2e-16^a^

**Burst size**

Initial hosts, day 4 PI Common garden hosts, day 4 PI

tr: χ^2^(1)=12.73 p=0.0004^a^ tr: χ^2^(1)= 0.058 p= 0.8103 ^a^

**Gametocyte dynamics**

Initial hosts, day 2-4 PI Common garden hosts, day 1-16 PI

tr*dayPI: χ^2^(2)=9.92 p=0.0070 ^a^ tr*dayPI: χ^2^(15)= 45.24 p=7.03e-05^a^

Common garden, day 2-16 PI

tr*dayPI: χ^2^(14)= 13.79 p= 0.4654

tr: χ^2^(1)= 8e-04 p= 0.9770

dayPI: χ^2^(14)= 630.4 p< 2.2e-16^a^

**Conversion rate**

Initial hosts Common garden, day 1-14 PI

not done tr*dayPI: χ^2^(13)= 6.43 p= 0.9292

tr: χ^2^(1)= 0.176 p= 0.6748

dayPI: χ^2^(13)= 236.69 p< 2.2e-16^a^

^a^ included in the final model
